# Environmental Exposure, Obesity, and Parkinson’s Disease: Lessons from Fat and Old Worms

**DOI:** 10.1289/ehp.1002522

**Published:** 2010-08-25

**Authors:** Layla Aitlhadj, Daiana Silva Ávila, Alexandre Benedetto, Michael Aschner, Stephen Richard Stürzenbaum

**Affiliations:** 1 King’s College London, Pharmaceutical Science Division, London, United Kingdom; 2 Department of Pediatrics, Vanderbilt University Medical Center, Nashville, Tennessee, USA

**Keywords:** *C. elegans*, heavy metals, neurodegeneration, obesity, Parkinson’s disease, serotonin

## Abstract

**Background:**

A common link has been exposed, namely, that metal exposure plays a role in obesity and in Parkinson’s disease (PD). This link may help to elucidate mechanisms of neurotoxicity.

**Objective:**

We reviewed the utility of the nematode, *Caenorhabditis elegans*, as a model organism to study neurodegeneration in obesity and Parkinson’s disease (PD), with an emphasis on the neurotransmitter, dopamine (DA).

**Data sources:**

A PubMed literature search was performed using the terms “obesity” and any of the following: “*C. elegans*,” “central nervous system,” “neurodegeneration,” “heavy metals,” “dopamine” or “Parkinson’s disease.” We reviewed the identified studies, including others cited therein, to summarize the current evidence of neurodegeneration in obesity and PD, with an emphasis on studies carried out in *C. elegans* and environmental toxins in the etiology of both diseases.

**Data extraction and data synthesis:**

Heavy metals and DA have both been linked to diet-induced obesity, which has led to the notion that the mechanism of environmentally induced neurodegeneration in PD may also apply to obesity. *C. elegans* has been instrumental in expanding our mechanism-based knowledge of PD, and this species is emerging as a good model of obesity. With well-established toxicity and neurogenetic assays, it is now feasible to explore the putative link between metal-and chemical-induced neurodegeneration.

**Conclusions:**

One side effect of an aging population is an increase in the prevalence of obesity, metabolic disorders, and neurodegenerative orders, diseases that are likely to co-occur. Environmental toxins, especially heavy metals, may prove to be a previously neglected part of the puzzle.

Obesity is defined as the abnormal or excessive accumulation of fat that presents a risk to health. Globally there are more than 300 million adults who are obese [World Health Organization (WHO) 2010] and 42 million children who are overweight (WHO 2010). Obesity is caused by a complex interplay of multiple factors (environmental and genetic)that can be ascribed to an imbalance in energy homeostasis under the control of the central nervous system. Although evidence for a role of the hypothalamus in obesity was derived from lesion experiments conducted > 50 years ago (Kennedy 1953), many gaps still remain in our knowledge of hypothalamic fat regulation. Recent evidence has implicated obesity in conferring a greater susceptibility to the adverse effects of environmental exposure (Chung and Yoon 2008). Conversely, there is evidence to suggest that the neuronal damage caused by environmental exposure can induce obesity (Edwards and Myers 2008). In the past, obesity as a result of environmental exposure was largely overlooked because of the commonly held belief that increases in obesity after the industrial revolution were due solely to changes in eating habits. However, concurrent with the industrial revolution was the mobilization of and increased exposure to heavy metals such as lead and mercury (Hg). To our knowledge, no epidemiological studies have provided a direct link between heavy metal exposure and obesity in humans. However, circumstantial evidence was offered by Turunen et al. (2008) who identified a correlation between high fish consumption, increased concentrations of environmental toxins in serum, and increased obesity. Furthermore, epidemiological evidence has suggested that obesity increases the susceptibility of fine metal particulate–induced cardiovascular effects (Chen et al. 2007). Because the intricate dissection of the underlying mechanisms within complex mammalian systems can be challenging, scientists have found that using simple eukaryotic invertebrate models, such as the nematode *Caenorhabditis elegans*, have proved to be tangible alternatives (Lant and Storey 2010).

## *C. elegans*—general concepts

*C. elegans* is a small, free-living, soil-dwelling nematode that requires a humid environment, atmospheric oxygen, and bacteria as a food source (Byerly et al. 1976). *C. elegans* is predominantly a self-fertilizing hermaphrodite (and thus in essence clonal), has a short life span of approximately 18–20 days, a rapid generation time (3 days at 22°C), and a large brood size (around 300 progeny per hermaphrodite). Its transparency allows the noninvasive visualization of cellular structures and green fluorescent protein (GFP)–tagged transcripts and proteins, even within single neurons. All neurons and synapses develop stereotypically and have been mapped by 3-dimensional reconstruction of transmission electron microscopy (TEM) serial sections (White et al. 1986). Furthermore, the genome of *C. elegans* has been fully sequenced and is highly conserved with that of mammals (60–80% homology) (*C. elegans* Sequencing Consortium 1998). Because of the detailed knowledge of its genomic architecture and the ease of genetic manipulation, scientists have generated thousands of knockout alleles and transgenic strains that has permitted detailed analyses of mechanistic neurotoxicology.

The nervous system is the most complex organ in *C. elegans.* The hermaphrodite comprises 302 nerve cells and 56 epithelioglial cells, which together equate to 37% of all somatic cells (Bargmann 1998). Nerve cells are concentrated in the circum-pharyngeal nerve ring in the ventral nerve cord and in the tail, with most of the sensory neurons located in the head region. A male *C. elegans* (a rare sex, with a frequency of 0.5%) has 89 additional neurons, most of which are posterior sensory and motor neurons (Sulston and Horvitz 1977; Sulston et al. 1980) that are implicated in male mating behavior (Whittaker and Sternberg 2009). The worm contains neurotransmitters inherent in the mammalian nervous system, including serotonin, dopamine (DA), γ-aminobutyric acid (GABA), glutamate, and acetylcholine, as well as numerous neuropeptides (Bargmann 1998). Neurotransmitter-specific transporters (membrane and vesicular) and receptors (including G-protein coupled) are also highly conserved with their mammalian counterparts (Bargmann 1998). The *C. elegans* nervous system is responsible for the control of all basic functions in the worm, and it regulates development, feeding, movement, metabolism, and reproduction (Chatterjee and Sinha 2008; Rankin 2002). Accordingly, alterations in any neurotransmitter system cause disturbances in specific functions commensurate with the affected type of neuron. Consequently, *C. elegans* is well recognized for its utility as an animal model for deciphering mechanisms of neurodegeneration.

This review aims to provide an authoritative overview demonstrating how *C. elegans* can be exploited as a model to study obesity and neurodegeneration, with a particular focus on environmental toxins and their role within the dopaminergic system. First, we describe the use of *C. elegans* as a model of obesity and the parallels to mammalian feeding and fat regulation. Next, we highlight the evidence of neurodegeneration in obesity and the impact of obesity on neurodegeneration. We then examine how the nematode has been used to study neurodegeneration in the Parkinson’s disease (PD) model using DA as the main neurotransmitter. Finally, we investigate the effects of heavy metals and chemicals on neuronal degeneration.

## *C. elegans*—the plus-size model

**Figure 1:**
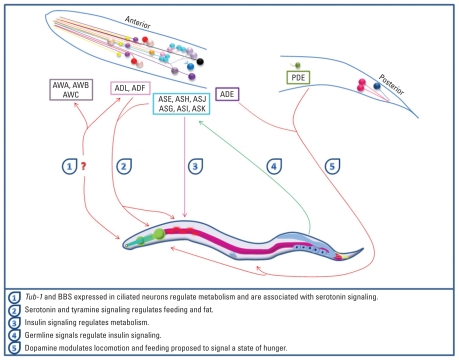
Neuronal control of feeding behavior and fat regulation in the nematode. Some of the neurons in the head region are exposed to the environment and integrate nutritional signals (olfactory, gustatory, and chemosensory) to coordinate numerous peripheral responses such as fat metabolism and reproduction. Peripheral sites of fat regulation are also able to feed back to the neurons to promote or inhibit neuroendocrine signals in neurons and peripheral sites. Colors of neurons in the nematode are matched to the key neurons annotated in the colored boxes. The question mark (1) indicates an unconfirmed hypothetical link. ADE indicates the anterior deirid; ADF, ADL, ASE, ASG, ASH, ASI, ASJ, and ASK are chemosensory neurons; AWA, AWB, and AWC are odorsensory neurons; BBS is a neuronally expressed gene associated with both adult and childhood obesity; PDE indicates the posterior deirid; and *tub-1* is isoform 1 of Tubby protein homolog. Adapted from Inglis et al. (2006) and Mak et al. (2006).

Although the nematode has long served as a model organism for a range of diseases, only recently has this species been considered relevant to obesity research. In 2003, Ashrafi et al. (2003) and McKay et al. (2003) independently described *C. elegans* as a useful model for gaining insights into the genetics of fat regulation. The advantage of having the fully sequenced *C. elegans* genome has permitted the identification of numerous genes encoding regulators of fat storage and transport as well as neuroendocrine regulators of fat and food-related behaviors, many of which have mammalian homologs (Mullaney and Ashrafi 2009). The core metabolic processes governing fat regulation are highly conserved, as is the lipid content of nematodes, which occurs primarily in the form of triglycerides, the major form of fat in foods (Ashrafi 2007; Burnell et al. 2005; Holt and Riddle 2003; McKay et al. 2003; Van Gilst et al. 2005; Wang and Kim 2003). As in mammals, fat levels in the nematode increase as a result of excess calories from sugars such as fructose and glucose, and the mechanism of this fat accumulation and utilization is regulated by a variety of well-preserved, albeit complex, transcriptional, translational, and posttranslational regulatory systems (Nomura et al. 2009) (Figure 1).

Despite these overwhelming analogies, there are some differences in worm fat regulation that arguably could be considered disadvantages. However, when treated correctly, these differences can be powerful tools in unraveling unique aspects of fat metabolism. For example, *C. elegans* are able to synthesize polyunsaturated fatty acids (PUFAs), whereas mammals must obtain these fats from their diet as essential fatty acids. This difference renders it possible to easily examine the roles of PUFAs through the use of genetic deletion techniques and separately from the complex signaling pathways activated by eicosanoids (derivatives of 20-carbon PUFAs involved in many processes, particularly the inflammatory response) in pathways not conserved in the nematode (Marza and Lesa 2006). Another striking difference between *C. elegans* and mammals is that the intestinal cells of worms serve as sites both of fat storage and metabolism, whereas mammals have dedicated adipocytes that store fat. In mammals, leptin secretion by adipocytes increases in response to excessive energy intake and acts on both appetite and peripheral tissues (Halaas et al. 1995; Shimabukuro et al. 1997). The lack of adipocytes and leptin in worms removes one of the complex layers involved in the centralized control of fat metabolism, thus making it easier to reveal the other conserved and underlying mechanisms of control. In addition, it has recently been demonstrated that Nile red staining of fat, a technique used to stain live worms and to ascribe a fat regulation role to > 400 genes, does not stain the major lipid stores in the worm (Ashrafi 2007; O’Rourke et al. 2009). Rather, the Nile red technique stains fats stored in lysosomal vesicles. It is therefore not representative of fixative staining techniques such as Sudan black and Oil Red O, which better represent results from biochemical analysis methods such as gas chromatography-mass spectrometry or visualization using coherent anti-stokes Raman scattering (Hellerer et al. 2007; O’Rourke et al. 2009). Although lipid accumulation in lysosomes is an important part of lipid metabolism and has a role in the pathology of diseases such as Tay Sachs and Niemann Pick, the data can be misleading if used incorrectly, that is, assuming that lipid accumulation is a measure of major fat storage, which can easily lead to numerous misinterpretations. Nonetheless, such lipid storage may be more important in worms because of the lack of adipocytes; hence, lysosomal staining should not be overlooked.

## Neuronal regulation of feeding

The nematode feeds continuously, but the feeding rate is not passive. Rather, it is modulated by food availability that is detected by the central nervous system. Similar to mammalian regulation of feeding, serotonin signaling in the worm plays an integral, albeit paradoxical, role. In mammals, both the rates of feeding and fat accumulation increase when serotonin levels are elevated. However, in *C. elegans*, increased serotonin signaling results in the reduction of fat accumulation while counterintuitively augmenting the feeding rate (Srinivasan et al. 2008). Although the ability of serotonin to control satiety and fat accumulation by two independent pathways is presented as a distinct feature of nematodes, evidence suggests that similar mechanisms also exist in mammalian systems. For example, tubby mutants in mice and nematodes (*tubby* and *tub-1*, respectively) display adult-onset obesity without exhibiting hyperphagia (Coyle et al. 2008; Mukhopadhyay et al. 2005). Because there is evidence that the Tubby protein can interact with G-coupled receptors, including the 5-HT2c (serotonin) receptor (Santagata et al. 2001), it is conceivable that there is a similar mechanism of independent fat and feeding regulation by serotonin that has yet to be fully revealed in mammals. This tubby model of obesity is different from that of *ob/ob* or *db/db* mice, mutant strains that follow the increased feeding and increased fat accumulation pattern (Campfield et al. 1995; Trayhurn and Fuller 1980). If such distinct mechanisms were found to occur in mammals, potential therapies that were independent of feeding habits could be targeted for further research. These therapeutic techniques, if effective, could prove to be particularly valuable in the treatment of obesity, especially considering the poor reputation of appetite-suppressing drugs (Williams 2010).

Satiety and hunger play important roles and together constitute the cornerstone for the prevention and treatment of obesity. Behavior reminiscent of satiety and hunger has also been identified in *C. elegans*. Under certain conditions, the nematode stops feeding and moving, a behavior termed “quiescence.” Because quiescence is dependent on food quality, nutritional signals from the intestine, and prior feeding history, quiescence is thought to indicate a state of satiety regulated by cyclic guanosine monophosphate and transforming growth factor β (TGF-β) (You et al. 2008), pathways whose functions in appetite control and metabolism have not yet been elucidated in mammals. However, TGF-β has also been shown to be elevated in the obese mouse models, *ob/ob* and *db/db*, which display increased feeding and obese phenotypes (Samad et al. 1997), thus suggesting a similar role for TGF-β in the appetite regulation of mammals.

In *C. elegans*, serotonin and tyramine are believed to act as signals for hunger; the latter modulates feeding and is a functional counterpart of norepinephrine (Roeder 2005). Tyramine signaling elicits increased food intake in the worm, a behavior normally caused by food deprivation. In the worm, as well as in mammals, the role of the neurotransmitters serotonin and norepinephrine has been explored, and targets for therapy have been attempted. However, the role of DA has only recently emerged in mammals, and few studies describe its role in obesity pathways. In *C. elegans*, DA is best known for mediating the slowing of movement that occurs when the animal encounters food, a behavior termed the “basal slowing response” (Sawin et al. 2000). DA is hypothesized to be released in response to food, and it acts on motor neurons to modulate the movement of the nematode (Chase and Koelle 2007). A recent study has shown that increasing DA signaling stimulated movement in *daf-2* dauers and dauerlike adults, but not in nondauer animals, thus suggesting that the reduction in insulin/insulin-like growth factor (IGF)-1 signaling is able to modulate the response of the animals to DA (Gaglia and Kenyon 2009). This altered response to DA is mediated in part by increased DAF-16/FOXO transcriptional activity in the nervous system. The notion that the insulin/IGF-1 signaling pathway can alter the activity of the DA pathway in *C. elegans* is compatible with the emerging idea that, in the mammalian brain, hormonal signals involved in energy balance, including insulin, can affect the DA reward pathway to modulate feeding behavior (Figlewicz et al. 2007). Food-restricted animals, which have lower circulating insulin levels, behave as though they have elevated DA signaling. This is analogous to norepinephrine in mammals, which, at levels of excess, mimics the perception of hunger (Figlewicz et al. 2007; Palmiter 2007). However, the mechanism by which insulin signaling acts at the cellular level to influence DA signaling remains unclear.

## Neurodegeneration and obesity—the chicken or the egg?

Because neuronal control is the central component of fat regulation, it is logical to assume that the integrity of this system (i.e., the functional components such as neurons) is of equal importance in maintaining energy homeostasis. In higher vertebrates, gross abnormalities in the brain structure and architecture, particularly in the frontal lobe, have been associated with obesity (Pannacciulli et al. 2006; Taki et al. 2008; Ward et al. 2005). Although it is unlikely that obesity is directly responsible for brain atrophy, there are several lines of evidence in mammals that demonstrate an association between increased adiposity and exacerbated neurodegeneration resulting from chemical induction as well as from Alzheimer’s disease (Chen and Lipton 2006; Moroz et al. 2008; Sriram et al. 2002). For example, Moroz et al. (2008) found that feeding a high-fat diet to mice for 16 weeks caused an increase in body weight in conjunction with type 2 diabetes (a common comorbidity of obesity) and a marginally reduced brain weight. Interestingly, however, these findings were associated with significantly increased levels of several substrates including tau, which is part of the pathology of many neurodegenerative diseases (Moroz et al. 2008). Another study by Sriram et al. (2002) used the neurotoxicant methamphetamine (METH), a well-characterized dopaminergic neurotoxicant that causes a marked decrease in striatal DA, tyrosine hydroxylase (TH), and DA transporter binding sites (Deng and Cadet 1999; Kogan et al. 1976; Sonsalla et al. 1989) to mimic some key features associated with the pathogenesis of PD. Their data indicated that the neurotoxic effects of METH in lean mice (decreased striatal DA and TH protein as well as an increase in glial fibrillary acidic protein) were exaggerated in the obese mice, thus implicating obesity as a risk factor for susceptibility to neurotoxic insult (Sriram et al. 2002). Furthermore, the administration of METH resulted in mortality in *ob/ob* mice but not in their lean littermates. In addition, the administration of METH resulted in the upregulation of the mitochondrial uncoupling protein-2 to a greater extent in the *ob/ob* mice than in the lean mice. This effect is known to reduce adenosine-5′-triphosphate (ATP) yield and to facilitate oxidative stress and mitochondrial dysfunction. In summary, the results implicate obesity as a risk factor associated with chemical-and possibly disease-induced neurodegeneration (Sriram et al. 2002).

Although neurodegeneration can easily be visualized in *C. elegans* by labeling neurons with fluorescent markers such as FITC (fluorescein isothiocyanate), DiI (1,1′-dioctadecyl-3,3,3′,3′,-tetramethylindo-carbocyanine perchlorate), DiO (3,3′-dioctadecyloxa carbocyanine perchlorate), and DiD (1,1′-dioctadecyl-3,3,3′,3′-tetramethylindodicarbocyanine perchlorate) (Tong and Burglin 2010), the direct role of adiposity on neurodegeneration has not been explored to date. Nevertheless, many conserved pathways relevant to obesity as well as neuronal cell death are present in the nematode, such as the p38 mitogen-activated protein kinase (Heida et al. 2010) and AKT signaling cascades, the ubiquitin-proteasome pathway (Doherty et al. 2008), and the oxidative stress response (Uttara et al. 2009), all of which have been found to cause neuronal injuries in the worm (Ayyadevara et al. 2008; Gami et al. 2006; Grad and Lemire 2004; Inoue et al. 2005; Tullet et al. 2008). In addition, it has been reported that obese humans and rodents display increased adipocyte apoptosis; further, apoptosis has been implicated in many neurodegenerative diseases including PD (Bharathi et al. 2006; Hirata 2002; Koh 2001; Ong and Farooqui 2005). The PD model for obesity in worms exhibits polyQ aggregation, which has been found to be toxic to neurons in worms, an effect similar to that in mammals. Additionally, in worms as in mammals, polyQ aggregation has been shown to be mediated by *daf-16* and the insulin-signaling pathway (another important pathway in obesity) (Voisine et al. 2007).

These findings support the notion that an environment of obesity aggravates neurodegeneration. However, there is evidence to suggest that the reverse is also true, that is, that neurodegeneration may lead to obesity. Examples of this include genes such as Ubb and tubby, which are both expressed in ciliated neurons. Noben-Trauth et al. (1996) and Ryu et al. (2008) found that defects in each of these genes lead to adult-onset obesity in mammals. Benzinou et al. (2006) found that another neuronally expressed gene, BBS, is associated with both adult and childhood obesity. Because the neurodegeneration occurs prior to the obese phenotype, it is likely that neuronal control of fat regulation is disrupted and leads to energy imbalance, thus resulting in adult-onset obesity, as opposed to the early-onset obesity exhibited by models such as ob and db. The worm has homologs of these three genes expressed in the ciliated neurons. These homologs show altered fat accumulation and altered longevity under certain conditions (Aitlhadj and Stürzenbaum 2010; Ashrafi et al. 2003; Mukhopadhyay et al. 2005; Pan et al. 2005). A partial list of neuronally expressed genes associated with increased fat accumulation when mutated is presented in Table 1. Regardless of the direction or initiation of obesity and neurodegeneration (cause or consequence), both conditions result in altered levels of neurotransmitters such as serotonin, DA, and norepinephrine which can cause neurotoxicity (e.g., serotonin syndrome), a vicious cycle that extends beyond both conditions and has far-reaching knock-on effects. The alteration of neurotransmitter levels results in disease phenotypes in both worms and mammals (Donohoe et al. 2006; Hapiak et al. 2009; Holson et al. 1994). In addition, the chronic elevation of serotonin signaling in the hypothalamus induces peripheral insulin resistance, consequently causing obesity in mammals (Belsare et al. 2010).

Despite evidence linking obesity to neurodegeneration, the relationship between these two conditions has become the focus of explorative research only recently; therefore, the underlying mechanisms remain unidentified. However, the role of the neurotransmitter DA is beginning to emerge. The next section aims to review our current knowledge regarding neurodegeneration in the PD nematode model and to demonstrate how this information can be used to help unravel the mechanisms underlying the neurodegeneration observed in obesity.

## *C. elegans* in Parkinson’s disease

PD is characterized by the progressive loss of dopaminergic neurons and manifests as muscle rigidity, tremors, and bradykinesia. PD affects DA-producing neurons in the brain, and the central tenet regarding the pathology of this disease holds that the loss of these neurons induces the symptoms of PD. DA is associated with motor activity and feelings of reward. However, recent evidence has highlighted that norepinephrine neurons also play a role in the etiology of PD. Norepinephrine is a neurotransmitter that controls attentiveness, emotions, sleeping, dreaming, and learning, but also acts as a hormone to increase heart rate and blood pressure, trigger the release of glucose and fatty acids from energy stores, and open up the air passages to the lungs. Although the precise etiology of the vast majority of PD cases remains elusive, it has been hypothesized that genetic and environmental factors are the causative denominators of the observed neurodegeneration. The *C. elegans* nervous system is limited to a mere 302 neurons. However, the diversity of classical neurotransmitters and neuropeptides is comparable to that of a vertebrate nervous system. The worm synthesizes DA and octopamine, the latter of which is a neurotransmitter considered to be an invertebrate counterpart to norepinephrine (Roeder 2005). Exogenous octopamine inhibits egg laying and pharyngeal pumping and acts antagonistically to serotonin, which stimulates pharyngeal pumping and egg laying (Horvitz et al. 1982; Niacaris and Avery 2003). The field of research exploring octopamine-driven neurotoxicity in *C. elegans* is still in its infancy, but a significant volume of work has focused on DA in PD.

## Parkinson’s disease genes

Studies with *C. elegans* orthologs of human genes linked to familial PD have led to novel insights into DAergic neurodegeneration. The worm genome encodes genes for *Parkin, PARK 9*, *UCHL-1* (ubiquitin carboxy-terminal hydrolase L1), *DJ-1*, *NURR1* (NUR-related factor 1), *PINK1* (PTEN-induced kinase 1), and *LRRK2* (leucine-rich repeat kinase 2)*,* but notably lacks α-synuclein (Benedetto et al. 2009; Gitler et al. 2009; Hamamichi et al. 2008; Sakaguchi-Nakashima et al. 2007; Samann et al. 2009; Ved et al. 2005). α-synuclein is a polypeptide with a propensity toward intracellular accumulation into inclusions referred to as Lewy bodies, which have been invoked as initiators of PD (Singleton et al. 2003). In *C. elegans*, overexpression of human α-synuclein causes significant loss of DAergic neurons (Hamamichi et al. 2008; Lakso et al. 2003). High-throughput RNAi-based screening in *C. elegans* revealed five neuroprotective genes that affect α-synuclein aggregation. The gene products include an ubiquitin-activating E1 enzymelike protein and a protein involved in lysosomal trafficking, VPS41. Notably, overexpression of VPS41 was shown to cause decreased α-synuclein aggregation and protection from DAergic degeneration induced by α-synuclein (Hamamichi et al. 2008). Further studies investigating the neuroprotective effect of VPS41 against α-synuclein–induced DAergic degeneration demonstrated that the protein acts by reducing cell death, attenuating the apoptotic cascade, and reducing the accumulation of detergent-insoluble, high-molecular-weight forms of α-synuclein (Ruan et al. 2010). Interestingly, the levels of α-synuclein are also relevant to obesity, as increased α-synuclein is reportedly consistent with increased leptin and obesity (Oort et al. 2008).

Several scientists have demonstrated that the genetic modulation of *parkin (pdr-1)*, *dj-1, pink-1*, and *lrk-1* (the gene that encodes LRRK2) disrupts mitochondrial function in *C. elegans* (Samann et al. 2009; Ved et al. 2005). This mitochondrial dysfunction is believed to play a role in triggering DAergic degeneration. For example, Ved et al. (2005) showed that the loss of function of *dj-1* caused a decrease in oxygen consumption and survival after exposure to rotenone—effects that were reverted by antioxidants. Similarly, *pink-1* knockout resulted in reduced mitochondrial cristae length in muscle and neuronal cells and defects in axonal outgrowth of a pair of canal-associated neurons, whereas *lrk-1 (tm1898)* mutants displayed an aberrant axon pathfinding (Samann et al. 2009). Of particular importance, this same study demonstrated an antagonistic role of PINK-1 and LRRK-2 in cellular functions. Samann et al. (2009) demonstrated that mutations in both genes suppress the phenotypic effects observed in the respective single mutants. Furthermore, *parkin* (*pdr-1*) deletion caused reduced levels of high-molecular-weight ubiquitin conjugates. This represents the first *in vivo* evidence that loss of a *parkin* homolog affects the ubiquitin proteasome system (UPS), effects that could not be observed in the mouse and *Drosophila* models (Ved et al. 2005). The UPS is the major route through which intracellular proteolysis is regulated. Reduced levels of ubiquitin have also been observed in obese patients (Chang et al. 2009), thus lending support to the notion that features of neurodegeneration are apparent in the obese. Taken together, these studies demonstrate that neurotoxicity studies at the genetic level in *C. elegans* are tremendously valuable in deciphering mechanisms that underlie mammalian DAergic degeneration.

## The dopaminergic system

DA and norepinephrine are monoamine neurotransmitters and are derived from tyrosine. In mammals, DA is produced by the conversion of the amino acid, tyrosine, to 1-dihydroxyphenylalanine (l-DOPA), a reaction mediated by TH, followed by l-DOPA metabolism to DA by the aromatic amino acid decarboxylase (AADC). Cytosolic DA is rapidly packaged into synaptic vesicles by a vesicular monoamine transporter (VMAT), where DA is stored and secreted upon neuronal depolarization. After exocytosis to the synaptic cleft, DA binds to its pre-and postsynaptic receptors (D1–D5). The DAergic signal is terminated by presynaptic reuptake, which is mediated by the dopamine transporter (DAT), followed by its inactivation by monoamine oxidase (MAO) or the catechol-*O*-methyl transferase. In a parallel pathway, DA can also be converted to norepinephrine by DOPA β-hydroxylase, which binds to the adrenergic receptors α1, β1, and β2. The norepinephrine signal is terminated by presynaptic reuptake, which is mediated by the norepinephrine transporter, followed by inactivation by MAO.

**Figure 2:**
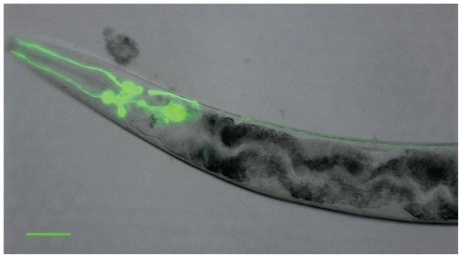
Pdat-1: GFP (green fluorescent protein) expression in the DAergic head neurons in *C. elegans*. Bar scale represents 50 μM.

DA modulates movement, defecation, egg laying, and food sensation in the worm (Schafer and Kenyon 1995; Weinshenker et al. 1995). It was initially detected by means of formaldehyde-induced fluorescence in eight sensory neurons of the hermaphrodite adult: four cephalic (CEP) neurons, two anterior deirid (ADE), and two posterior deirid (PDE) neurons (Sulston et al. 1975) (Figure 2). Additional neurons were noted in the male nematode, specifically in six rays of the male tail, referred to as the R5A, R7A, and R9A pairs of neurons. All of these neurons are believed to be mechanosensory, and the ablation of these cells causes defects in the ability of the animal to sense or respond to environmental changes (Duerr et al. 1999; Hills et al. 2004; Sawin et al. 2000). The presence of DA, including its precursors and metabolites, has been confirmed in *C. elegans* extracts by chromatography and spectroscopy (Wintle and Van Tol 2001), demonstrating that DA levels in synaptic vesicles are remarkably similar to those in mammalian neurons (Rand et al. 1998). Furthermore, the full requisite machinery for DA synthesis, storage, release, transport, and binding in *C. elegans* has been uncovered and systematically characterized (McDonald et al. 2006; Weinshenker et al. 1995; Wintle and Van Tol 2001). Given the parallels in DAergic homeostasis between *C. elegans* and mammals, the nematode has proved an invaluable model system for understanding human diseases that implicate abnormalities in DAergic function.

*C. elegans* forward genetics have highlighted several genes that encode proteins associated with DAergic function. The first DA-related loss-of-function mutation identified affected *cat-1*, a gene with 47% and 49% homology to human VMAT-1 and VMAT-2, respectively (Duerr et al. 1999). *Cat-2* is the *C. elegans* homolog of mammalian TH, and it is expressed in all DAergic neurons, as demonstrated by a GFP construct fused to the *cat-2* promoter (Lints and Emmons 1999). An aromatic AADC homolog (*bas-1*) has also been reported as an ortholog of the human guanine triphosphate (GTP) cyclohydrolase I gene, which, in mammalian systems, expresses a protein involved in the regulation of TH activity, namely *C. elegans cat-4* (Loer and Kenyon 1993). Furthermore, DA receptor genes have been identified as four mammalian homologs, *dop-1*, *dop-2*, *dop-3*, and *dop-4* (Chase and Koelle 2007). Finally, the DAT-1 transporter has been characterized using both genetic and pharmacological approaches (Jayanthi et al. 1998).

## Neurodegeneration by toxic chemicals

Most animal models for DAergic neurodegeneration are based on exposure to the neurotoxins 1-methyl-4-phenyl-1,2,3,6-tetrahydropyridine (MPTP) and 6-hydroxydopamine (6-OHDA), which chemically ablate DAergic neurons (Gerlach and Riederer 1996; Glinka et al. 1997). 6-OHDA and the reactive metabolite of MPTP, MPP^+^ (1-methyl-4-phenylpyridinium), selectively accumulate in DAergic neurons, causing increased reactive oxygen species (ROS) generation and/or mitochondrial dysfunction, thereby inducing neuronal damage and cell death (Glinka et al. 1997; Javitch et al. 1985).

As in mammals, exposure to 6-OHDA causes specific degeneration of DAergic neurons in *C. elegans*, as revealed by the dose-dependent decrease in the fluorescence signal in the P_dat-1_::GFP strain and also by the observation of pathological changes in TEM worm sections (Nass et al. 2002). The *C. elegans* platform has been highly instrumental in establishing that the presence of DAT-1 expression is essential for 6-OHDA–induced DAergic toxicity, as loss of *dat-1* (*ok157*) function renders the DAergic neurons insensitive to 6-OHDA (Nass et al. 2002). Interestingly, the 6-OHDA model has been successfully used in screens to identify not only genetic but also pharmacological suppressors of DAergic toxicity. The human DAT-1 antagonists imipramine, nisoxetine, and amphetamine, as well as the DA receptor antagonists bromocriptine, quinpirole, ranclopride, and SCH23390, have been shown to effectively protect against 6-OHDA neurotoxicity in *C. elegans*. These findings have established a valid rationale for future studies with these compounds to further elucidate their potential therapeutic modalities for the treatment of PD in humans (Marvanova and Nichols 2007; Nass et al. 2002). Hyperphagia and obesity can be induced experimentally in rodents by the microinjection of 6-OHDA into the ventral noradrenergic bundle to interrupt efferent catecholaminergic pathways to the hypothalamus. This interruption is thought to be due to decreased leptin and increased neuropeptide Y signaling (Kalra et al. 1998).

MPTP is highly lipophilic and readily crosses the mammalian blood–brain barrier and cell membranes. Intracellularly, it is metabolized to the active toxic metabolite MPP^+^. This polar molecule is secreted via the extraneural monoamine transporter (Russ et al. 1996) and actively taken up by DAergic cells via DA transporters (Javitch et al. 1985). In the mitochondria of DAergic neurons, MPP^+^ inactivates complex I of the respiratory chain (Mizuno et al. 1988), decreasing ATP levels and increasing free radical production, thus leading to DAergic neurodegeneration. Likewise, *C. elegans* treated with MPTP/MPP^+^ respond by significantly reducing mobility, which is associated with the specific degeneration of DAergic neurons (Braungart et al. 2004). Analogous to the findings with 6-OHDA (Nass et al. 2002), MPP^+^-induced DAergic neurodegeneration in *C. elegans* appears to be a caspase-independent cell death pathway, suggesting that caspase inhibitors may not be effective in rescuing DA neurons from cell death (Pu and Le 2008). It has also been reported that the loss of function of *vha-12*, a gene involved in necrosis-mediated neurodegeneration that is also expressed in DAergic neurons, leads to a hyposensitivity of *C. elegans* to MPP^+^, suggesting that this neurotoxin also triggers the activation of necrotic cell death pathways (Pu and Le 2008). Vha-12 is predicted to bind ATP; therefore, the consequences of MPP^+^ hyposensitivity could result in an altered energy homeostasis, an effect that would have implications for obesity.

In addition to chemical exposure, heavy metal exposure can cause neurodegeneration. Using a specific fluorescent marker to label the AFD sensory neuron (P*gcy-8*::GFP) revealed that Hg, copper (Cu), silver, and chromium cause a reduction in the relative intensities of cell bodies in AFD neurons, which regulate feeding via serotonin and tyramine signaling (Figure 1). Metals such as manganese (Mn) (Aschner et al. 2007; Benedetto et al. 2009; Guilarte 2010), vanadium (Afeseh Ngwa et al. 2009; Avila-Costa et al. 2004), and Cu (Wang et al. 2009; Wimalasena et al. 2007; Yu et al. 2008), as well as pesticides such as paraquat (Andersen 2003) and rotenone (Ishiguro et al. 2001) and bacterial toxins such as epoxomicin and lactacystin (McNaught et al. 2004; Schapira et al. 2006; Zeng et al. 2006) are all able to alter optimal DAergic function in mammals. The mechanism(s) that underlie the neurotoxicity of these toxicants involve mitochondrial dysfunction in DA-producing neurons, associated with energy depletion, increased ROS production, and cell death by apoptotic and/or necrotic pathways (Benedetto et al. 2009; Ishiguro et al. 2001; Lotharius et al. 1999). These are the same pathways thought to play a role in the neurodegeneration observed in obesity.

Mn is an essential trace metal necessary for normal brain development and for the optimal functioning of multiple enzymes, including Mn-superoxide dismutase and glutamine synthase (Brock and Walker 1980; Takeda and Avila 1986; Wedler and Denman 1984). Nevertheless, high levels of exposure to Mn in human occupational cohorts (mining, smelting, etc.) and in patients with cirrhosis undergoing total parenteral nutrition, have been shown to cause extrapyramidal symptoms that include rigidity, tremor, dystonic movements, and bradykinesia (Aschner et al. 2007; Dobson et al. 2004; Erikson et al. 2007). Researchers have demonstrated that Mn targets DA-rich areas in the brain, particularly in the basal ganglia (Aschner et al. 2007; Au et al. 2008). Mn can readily oxidize DA, thus generating the reactive metabolite leukoaminochrome o-semiquinone, which is highly toxic to DAergic neurons (Díaz-Véliz et al. 2004; Graumann et al. 2002). Nevertheless, despite the plethora of studies in mammalian models (Aschner et al. 2007; Burton and Guilarte 2009; Dobson et al. 2004), much of the insight on its preferential uptake and targeting of DAergic neurons has been derived from studies in *C. elegans*. For example, it has been shown only recently that the presence of DA is a requisite for the Mn-induced neurodegeneration of DAergic neurons *in vivo*. Making use of various genetic strains of *C. elegans* in conjunction with the fluorescent tagging of different classes of neuron types, Benedetto et al. (2010) demonstrated that Mn causes a dose-dependent degeneration of DAergic neurons. This effect was specific to DAergic and absent from GABAergic, serotononergic, or glutamatergic neurons. Furthermore, Mn was shown to cause the significant shortening or disappearance of DAergic neuronal extensions at low levels of exposure and eventually neuronal death, which was characterized by shrinkage of the cell body and the complete loss of GFP in P*dat-1*::GFP worms at higher levels of Mn exposure. A novel finding in the *C. elegans* model was that the Mn-induced DAergic degeneration required the presence of the reuptake transporter DAT-1, as *dat-1* loss of function abolished the Mn-induced DAergic GFP-fluorescence loss in the *dat-1*; Pdat-1::GFP worms (Benedetto et al. 2010). Conversely, animals lacking DAT-1 showed higher susceptibility to Mn toxicity than any other homozygous mutant tested, as observed with MPP^+^ exposure (Pu and Le 2008), suggesting that tissues other than those of the DAergic system were affected. This is consistent with a recent finding in mammals, which indicates that norepinephrine plays an important role in PD (Rommelfanger and Weinshenker 2007). Interestingly, Mn toxicity was shown to be prevented by the loss of TH/CAT-2 function in the double knockout strain, *cat-2(e1112); dat-1(ok157),* establishing that DA synthesis is required for DAT-1–dependent Mn-induced toxicity. It was further demonstrated that the absence of VMAT2/CAT-1 in *cat-1(e1111)* mutants, where DAergic neurons are unable to release DA at the synaptic cleft, resulted in an increased tolerance to Mn exposure, indicating that extracellular DA, but not intracellular DA, is involved in Mn toxicity. Corroborating this finding, the loss of function of the three DA receptors led to increased extracellular DA, which exacerbated Mn toxicity (Benedetto et al. 2010). These results were consistent with the extracellular Mn-induced oxidation of synaptic DA-generating ROS, as indicated by the increased fluorescence of the 2′7′-dichlorodihydrofluorescein diacetate probe and the increased cellular lipid oxidation inferred from variations in isoprostane levels (Benedetto et al. 2010).

Finally, additional experiments uncovered two more antagonistic mediators of the Mn-induced DA and DAT-1–dependent toxicity, namely, the dual oxidase BLI-3, an extracellular enzyme involved in the dityrosine cross-linking of collagen for cuticle formation in the worm, and SKN-1, the ortholog of the mammalian NRF-2 (nuclear factor-2 erythroid 2-related factor-2) responsible for the regulation of the expression of antioxidants (An and Blackwell 2003). The *bli-3(e767)* mutant strain exhibited hyperresistance to Mn exposure and failed to show any increase in ROS levels upon increasing levels of Mn exposure, and the combined exposure to Mn and exogenous DA did not affect the sensitivity of the worms to Mn, all results that have not been shown to occur in wild-type worms. These observations suggest that this dual oxidase mediates, at least partially, the Mn-induced and DA-dependent DAergic neurodegeneration. Conversely, *skn-1* mutants displayed increased sensitivity, while SKN-1 overexpressing worms were found to be hyperresistant to Mn exposure; further, SKN-1::GFP revealed an Mn-induced change in the nuclear localization of SKN-1 in the ASI pair of neurons (Benedetto et al. 2010), which are involved in the regulation of aging in *C. elegans* (An and Blackwell 2003; Tullet et al. 2008).

The sensitivity of DAergic neurons in *C. elegans* to Mn exposure is also reflected by the presence of Mn-specific homologs to the mammalian NRAMP/divalent metal transporter (DMT), referred to in the nematode as SMF. Translational GFP fusions for the three transporter isoforms, SMF-1, SMF-2, and SMF-3, revealed tissue-specific localizations of these transporters in the worm. SMF-1 and SMF-3 localize in the intestinal epithelium, whereas SMF-2 localizes in the pharyngeal epithelium, suggesting different roles for these transporters in relation to Mn uptake (Au et al. 2009). Furthermore, SMF-1 and SMF-2 were found to be expressed *ex vivo* in *C. elegans* DAT-1–expressing neurons, and the deletion of the *smf-1* gene attenuated the DAergic neurodegeneration caused by Mn (Settivari et al. 2009). Moreover, the loss of function of *smf-1* and *smf-3* caused a significant decrease in Mn accumulation and consequent increased survival after exposure to the metal, establishing that Mn uptake and toxicity via DMTs are conserved from nematodes to humans (Au et al. 2009). Taken together, Mn studies in *C. elegans* demonstrate that a single acute treatment at the first larval stage led to both developmental and aging defects, providing a link between early acute metal exposure, neurodegeneration, and longevity.

It has been proposed that environmental exposure during initial periods of the neural development may increase the susceptibility of the central nervous system to further exposures or increase the risk of developing neurodegenerative disorders, such as PD (Cory-Slechta et al. 2005). The short life span of *C. elegans* makes it a valuable model for exerting toxic insults early on during development and allowing the observation of effects within a short time span.

## Conclusions

The abundance of chemicals and heavy metals in the environment has led to human exposure via water, food, and air. Many of these substances are able to cross the blood–brain barrier, thus presenting a neurotoxic threat. Several neurodegenerative diseases have become more prevalent because of the increasing aging population. Therefore, neurotoxic poisoning is likely to co-occur with age-associated neurodegenerative diseases, with each condition potentially exacerbating the other. We have used the example of PD as an age-associated neurodegenerative disease because the incidence for PD increases rapidly in the population cohort exceeding 50 years of age. Although the precise etiology of the vast majority of PD cases remains elusive, there is evidence to show that heavy metals and toxic chemicals such as cadmium and paraquat can induce both the dopaminergic and noradrenergic neuronal degeneration characteristic of PD. However, environmental toxins have also been implicated in a wide range of other diseases, including obesity. As obesity is an epidemic that is ultimately regulated by the central nervous system, it is conceivable that environmental toxins act via mechanisms similar or identical to those in PD. For example, neurons affected by neurotoxins in PD contain DA and norepinephrine, both of which also play a role in the regulation of obesity. In addition, mitochondria are central to metabolism and represent a target to several environmental toxins that appear to contribute to the development of PD and obesity in response to environmental toxins by inducing apoptosis. The mitochondrial structure and function in *C. elegans* are similar to their mammalian counterparts, with many of the nuclear and mitochondrial encoded genes being highly conserved. A plethora of literature exists describing the role of serotonin in the regulation of both feeding and fat. However, relatively few studies have been carried out regarding the other neurotransmitters, largely because their role has emerged only recently. Deciphering the intricate interplay of neuronal control and degenerative diseases and obesity is a complex and multifaceted process. Genes do not work in isolation to generate particular phenotypes; rather, they interact with other genes and are influenced by the environment; therefore, using whole-animal invertebrate models such as *C. elegans* have proved to be particularly useful in these ongoing and important endeavors. One caveat of using an evolutionary distant soil invertebrate to model human disease is the notion that nematodes, like earthworms, are soil-dwelling organisms and thus it is conceivable that selection pressure (due to direct exposure to soil pollution) may have resulted in the rapid evolution of (distinct) toxicological response pathways (Hughes et al. 2009; Stürzenbaum et al. 2001, 2009; Zeitoun-Ghandour et al. 2010). However, even though some obstacles remain, the association between neurodegeneration in both PD and obesity, with a potential link to environmental toxins, is evident and highly relevant. The power of this simple worm to be predictive of mammalian systems has already resulted in the identification of many genes shown to be important in the etiology of various disease models and shows promise as an invaluable tool for medium and high-throughput toxicological screening. If forthcoming information is extrapolated correctly, the humble worm can teach us valuable lessons.

## Figures and Tables

**Table 1:** Partial list of neuronally expressed mutant alleles that exhibit increased fat accumulation.

Gene	Human ortholog	Neuronal expression
*rpy-1*	Isoform 1 of 43 kDa receptor-associated protein of the synapse	DA, VD, AS, VB, DB
*glr-7*	Glutamate receptor, ionotropic, kainate 3, isoform CRA_a	I3, I2, I6, MI, NSM, I1
*try-10*	Chymotrypsin-like elastase family member 1	Amphids and phasmids
*nhr-178*	Isoform 2 of Nuclear receptor subfamily 2 group C member 1	Amphids and phasmids
*acs-2*	Long-chain fatty acid acyl-CoA ligase	Several neurons
*uvt-6*	Somatostatin receptor type 4	Head and tail neurons
*T04C9.1*	Rho GTPase-activating protein 10	Head and tail neurons
*tub-1*	Isoform 1 of Tubby protein homolog	ASI, ADL, ASK, AWB, ASH, ASJ, PHA and PHB and PDE
*bbs-1*	Isoform 2 of Bardet-Biedl syndrome 1 protein	Head and tail neurons and mid-body PDE
